# Brainstem anesthesia after retrobulbar block under brief analgosedation: Evidence for the underlying patho-mechanism

**DOI:** 10.1177/11206721251333266

**Published:** 2025-04-16

**Authors:** M. A. Thomasius, M. Menghini, J. Breckwoldt

**Affiliations:** 1 Department of Anesthesiology and Perioperative Medicine, University Hospital of Zurich, Zurich, Switzerland; 2 Department of Ophthalmology, 30721 Ospedale Regionale di Lugano, Lugano, Switzerland

**Keywords:** Retinal detachment, retrobulbar block, brainstem anesthesia, emergency, perinterventional risks

## Abstract

**Background:** Retrobulbar block is a popular regional anesthetic technique in modern eye surgery due to its excellent anesthetic properties and the provision of globe akinesia. Severe complications including inadvertent subarachnoidal injection, expulsive retrobulbar hemorrhage, and intoxication with local anesthetic, are very rare. However, most reports date back several decades, mechanisms of action are not fully understood, and in recent years the procedure has changed towards facilitating the retrobulbar injection by a brief analgosedation. We therefore describe a case with inadvertent brainstem anesthesia after retrobulbar block concealed behind an analgosedation and provide cCT (cranial computed tomography) images with characteristic pathological findings. **Therapy and outcome:** A man in his mid-60´s presenting with retinal detachment was scheduled for surgery. After uneventful retrobulbar injection under brief analgosedation, a severe increase of blood pressure and tachycardia occurred while unconsciousness (originally induced by analgosedation) persisted. Hemodynamic alterations were treated with betablockers and antihypertensive agents, and the patient was intubated and mechanically ventilated. The diagnostic workup revealed a dural fissure with intracranial air in the cCT-scan compatible with a perforation of the dura and accidental injection of local anesthetics into the subarachnoidal space. The patient was kept intubated on ICU throughout the respiratory depression and fully recovered without neurological deficits. The vitreoretinal procedure was performed under general anesthesia 36 h after the event. **Conclusion:** Albeit rare, inadvertent brainstem anesthesia remains a serious adverse event of retrobulbar block. As an important aspect, analgosedation may mask the typical clinical signs making the diagnostic work-up challenging. Furthermore, for the first time we present radiographic imaging findings providing insightful evidence for a possible mechanism of action. Serious complications, such as prolonged hypoxia with potential neurological damage, can successfully prevented by ensuring the presence of a fully equipped and skilled anesthetic team throughout the regional anesthetic procedure.

## Case presentation

A male in his mid-60´s presented with left eye retinal detachment scheduled for emergency vitrectomy in January 2020. Secondary diagnoses were a well-controlled arterial hypertension, and mild chronic obstructive pulmonary disease (GOLD 1). For anesthetic procedure a retrobulbar block combined with brief initial analgosedation was chosen.

After establishing routine monitoring of vital parameters (ECG, NIBP, pulse oximetry) and placing an intravenous line, the patient received pre-oxygenation (until reaching etO2 of 80%) and a bolus of 50 µg of fentanyl und 150 mg (2 mg/Kg) of thiopental according to house standard. Immediately after loss of consciousness, the surgeon applied the retrobulbar block. A blunt 23 G needle of 32 mm length was used to apply the local anesthetic solution (2.5 ml mepivacaine 2% plus hyalase 20:1 and 2.5 ml bupivacaine 0.5%; total volume 5 ml) via a transconjunctival access at the infero-temporal margin of the orbita. Neither blood nor cerebrospinal fluid could be aspirated during the procedure. A typical proptosis during injection, however, was not observed. Subsequently, occulopression was performed without causing subsequent bradycardia. Instead, after approximately one more minute we observed a raise in heart rate (103 bpm) and blood pressure (213/133 mmHg). While urapidil was prepared, hypertension increased to a value of 270/166 mmHg, and tachycardia reached 130 bpm. After two i.v. doses of 10 mg of urapidil, the systolic blood pressure fell below 200 mmHg while tachycardia persisted. Paralleling the hemodynamic crisis, we observed that the patient did not take up respiration again. In consequence, we started bag-mask ventilation with 100% oxygen supported by a ventilator. Right eye pupil reaction was unchanged. Left eye pupil had been pharmacologically dilated in preparation of the surgical procedure. An indirect light reaction could however not be observed in the right eye. At this point we established the working hypothesis of a toxic reaction to the local anesthetic, and immediately applied a bolus of 100 ml SMOF-Lipid solution followed by two ampoules with a total amount of 500 ml over 30 min according to the standard procedures for local anesthetic intoxication of the Swiss Society for Anesthesiology and Perioperative Medicine SSAPM.

**Figure 1. and 2.**
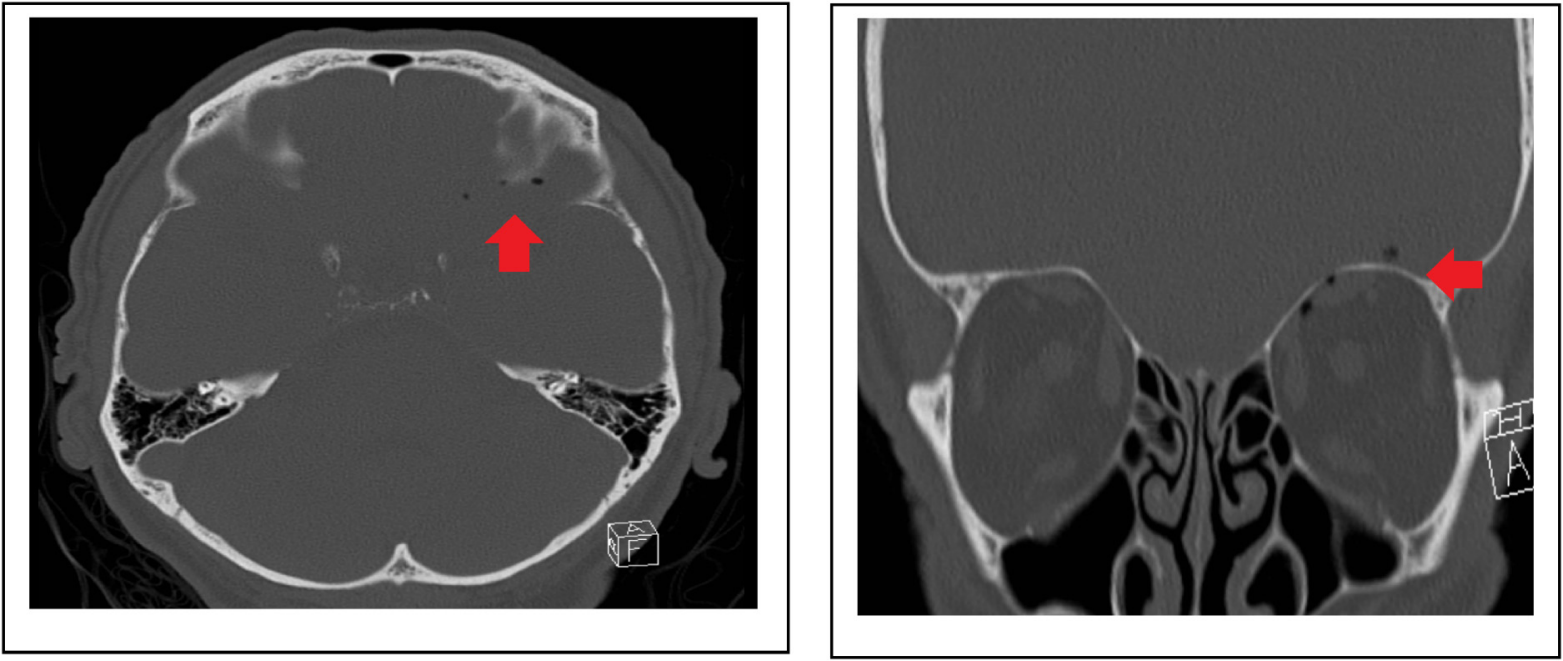
cranial CT showing intra-cranial air with suspicion of a small fissure at the upper orbita and a suspicion of blood in the left fronto-basal sulcus.

While there was persistent loss of consciousness and loss of spontaneous breathing, the working hypothesis extended to brainstem anesthesia. An arterial blood gas analysis excluded metabolic reasons of unconsciousness. The patient was then intubated and mechanically ventilated. We immediately conducted cranial CT scans (native, and with contrasting agent) to exclude further neurologic pathologies. The CT scans revealed intra-cranial air (see arrow in Figures 1 and 2) with a suspicion of a small fissure at the upper orbita and sparse blood in the left fronto-basal sulcus. The observed blood was accounted clinically insignificant. Signs of demarcated infarctions could be ruled out.

The patient was transferred to the ICU, and prophylactically treated with a single dose of intravenous Cefuroxime. Blood glucose levels and arterial blood gases were normal at any time. A couple of hours after the incident, the patient could be extubated without complications. On the following day, uneventful vitrectomy of the left eye was performed under general anesthesia. The patient was discharged without any deficits 48 h after the incident.

Informed consent has been obtained from the patient to use the data presented in this case report.

## Discussion

### Incidences of complications

Central nervous complications following retrobulbar anesthesia are very rare but have been reported in almost 30 cases. Most of these have been published in the past century.^1–19^ We found seven reports since 2010, and six more since 2000.^20–32^ Clinical manifestations reached from mild impairment like temporary gaze palsy up to blindness of the contralateral eye, dysphagia, respiratory depression, and cardiac arrest.^1,20^ The incidence of CNS complications was calculated between 0.27% und 0.79%.^2,3^ The frequency of respiratory depression after retrobulbar anesthesia was estimated at 0.03%.^4,5,6^ A study from 1987 analyzing 6’000 retrobulbar blocks found 16 cases (0.26%) in which neurologic symptoms indicated the local anesthetic had entered the intracranial space; one of these patients (0.02%) had suffered cardiac arrest.^1^ In our institution, we perform more than 1’000 retrobulbar blocks per anno without any similar complication reported so far. Table 1 in the appendix gives an overview of the characteristics of cases reported since 2000. The choice of the local anesthetic agent varies and depends on availability and pharmacological properties. The main consideration in combining two different local anesthetics target a combination of fast onset (Lidocaine and Mepivacaine) with sufficient duration of the effect (Bupivacaine and Ropivacaine).^33,34^

**Table 1.** Overview of cases reported since 2000.

Author, year	Type of Local anaesthesia	Volume applied	Substances applied	Clinical signs	Duration of clinical signs	Outcome (if known)
*Published after 2010*						
Yue-Lin Wang 2022^21^	Retro-bulbar	4 ml	2% Lidocaine/ 1% Ropivacaine	Lost consciousness, apneic	2 h	No residual defect
Nanda 2021^22^	Retro-bulbar	5 ml	0.5% Bupivacaine	Apneic in general anesthesia	Not mentioned	No residual defect
Kostadinov 2019^23^	Retro-bulbar	4 ml	2% Lidocaine/ 0.5% Levobupivacaine	Lost consciousness, hypotension, bradycardia, apneic	6 h (extubation)	No residual defect
Toleska 2016^24^	Retro-bulbar	2.5 ml	2% Lidocaine	Lost consciousness, hypotension, bradycardia	0.5 h	No residual defect
Dettoraki 2015^25^	Retro-bulbar	6 ml	0.75% Ropivacaine	generalized tonic-clonic seizures followed by transient contralateral hemiparesis	3 h	No residual defect
Jaichandran 2013^26^	Peri-bulbar	5 ml	2% Lidocaine/ 0.5% Bupivacaine	Contralat. third nerve palsy	*2 h*	Normal discharge, no residual defect
Aranda Calleja 2011^27^	Retro-bulbar	5 ml	2% Lidocaine/ 0.5% Bupivacaine	Agitation w. disorientation, zyanosis, hypertension	Not mentioned	No residual defect
*Published after 2000*						
Dahle 2007^28^	Retro-bulbar	4 ml	2% Lidocaine/ 0.75% Bupivacaine	Lost consciousness, Apnoe, hypertension, tachycardia, gerenalized seizure	24 h	No residual defect
Pragt 2006^29^	Retro-bulbar	5 ml	1% Ropivacaine	Localized convulsion of the ipsilateral Face, after that left-sided hemiparesis	1 h	No residual defect
Chhokra 2006^30^	Retro-bulbar	6 ml	4% Lidocaine/ 0.75% Bupivacaine	Apneic after 5 min, stayed unconscious	1.5 h	No residual defect
Gunja 2006^31^	Retro-bulbar	5 ml	2% Lidocaine/ 0.5% Bupivacaine	Foreign body sensation in the throat, Lost consciousness, apneic, hypotension, bradycardia	1 h	No residual defect
George 2005^32^	Retro-bulbar	9 ml + 5 ml	1% Lidocaine/ 0.375% Bupivacaine	Confusion, hearing loss, hypertension, tachycardia	1 h	No residual defect
Schönfeld 2000^20^	Retro-bulbar	5 ml	1% Mepivacaine	Dysphagia, nerve palsy, Apnea, Drop unconscious several minutes after injection	Several hours	No residual defect

Many surgeons prefer adding hyaluronidase, as hyaluronidase facilitates the spread of anesthetic to maximize its effect while reducing volume.^34^ Mentioning safety aspects, the dosage of applied local anesthetics in all the described cases were far below toxic threshold levels.^35,36^ In our case, for the maximum dose for Mepivacaine would have been 5 mg/kg^−1^ and for Bupivacaine 2 mg/kg^−1^.

### Diagnostic considerations

In our case, analgosedation masked the typical initial symptoms of either local aesthetic intoxication (such as metal taste, nausea, or impaired vigilance), or brain stem anesthesia (such as blindness, gaze palsy, shivering, dysphagia, loss of consciousness, respiratory depression, or respiratory arrest).^7,20^ In the light of an unconscious patient suffering severe hypertension coupled with tachycardia, a distraction between local anesthetic systematic toxicity (LAST) and brainstem anesthesia is challenging. In LAST, the initial hemodynamic reaction is related to the stimulation of the sympathetic nervous system. Local anesthetics can cause an increase in the release of catecholamines (like adrenaline) and inhibit their reuptake, leading to an overactive sympathetic response. This results in elevated heart rate (tachycardia) and increased blood pressure (hypertension).^37^ Because local anesthetics strongly affect mitochondrial metabolism, neurologic and cardiac dysfunctions occur later on with severe bradycardia and hypotension.^38^ There is limited data regarding the mechanism of hypertension and tachycardia after intrathecal injection. Hamilton described the patho-mechanism as a combination of vagal and carotid sinus reflex blockade.^16^ Another known description of sympathetic activation is due to a reaction of instant intracranial pressure rise. This reaction is described in animal studies: Intrathecal injections can increase cerebrospinal fluid (CSF) pressure with consecutive elevation of intracranial pressure (ICP). The rapid increase in ICP can trigger a reflex sympathetic response resulting in hypertension and tachycardia. This response is mediated by baroreceptors and chemoreceptors, which detect changes in pressure and chemical composition.^39^ Tachycardia would have also brought an anaphylactic reaction into discussion. However, hypertension and the lack of erythema or bronchospasm made this diagnosis very unlikely. Intracranial hemorrhage and stroke had been ruled out by CT scanning.

### What this case adds

The case presented, adds important aspects to the literature. One important point is that we were able to provide images of a morphologic correlate possibly explaining the patho-mechanism behind the clinical symptoms. CT scans clearly showed intra-cranial air and blood in the left fronto-basal sulcus which both could not be explained otherwise.

As a second important point, we add the conditions of brief analgosedation before the retrobulbar block. This technique is increasingly applied to enhance patient comfort. However, both local anesthetic intoxication, and brainstem anesthesia are primarily masked by the sedation.

In our case, the complications could be handled appropriately due to the presence of an anesthesia team. Standardized procedures including pre-oxygenation and close monitoring of vital signs were essential precautions leading to better safety margins when treating all the relevant conditions at the same time (hypertensive crisis, tachycardia, respiratory arrest). This preparation was a precondition for achieving a favorable outcome. In times of increasing ambulant practice and cost restraints, it is therefore essential to have a skilled anesthesia team available.

### Technical alternatives to the retrobulbar block

To avoid complications as the one described it is of interest whether adequate alternatives are available. One possible alternative is a peri-bulbar infiltration outside of the eye muscle cone. With this method, it takes considerably more time until full anesthesia is established, and bulbar akinesia is less reliably achieved as compared to the retrobulbar approach.^8,9^ Another alternative is subtenon anesthesia. For this technique, Tenon's capsule is opened by incision and the local anesthetic is injected after blunt dissection.^10,11^ Subtenon anesthesia leads to a success rate comparable to retrobulbar block (in terms of analgesia and bulbar akinesia). Drawbacks of this block include the necessity to open the conjunctiva with the risk of infection, and subconjunctival bleeding. Subtenon block is perceived as safer, because the blunt preparation makes bulbar injuries less likely and other severe complications such as brain stem anesthesia have not been reported. Accordingly, subtenon block is preferred in health services where an anesthesia team is not readily available. We therefore recommend subtenon anesthesia for such situations as the technique of choice.

## Conclusion

With this case, we present a rare but life-threatening complication of retrobulbar anesthesia for eye surgery. We report two important aspects for the first time. First, we provide imaging findings helping to better understand the patho-mechanism of action. Second, we describe this complication in the context of brief analgosedation that may mask many of the clinical signs. The presence of a skilled anesthesia team was essential for achieving a favorable outcome.

## References

[1] Nicoll JMV Acharya PA Ahlen K , et al. Central nervous system complication after 6000 retrobulbar blocks. Anesth Analg 1987; 66: 1298–1302. 3688501

[2] Brookshire GL Gleitsmann KY Schenk EC . Life-threatening complication of retrobulbar block. Ophthalmology 1986; 93: 1476–1478. 3808610 10.1016/s0161-6420(86)33543-7

[3] Rodman DJ Notaro S Peer GI . Respiratory depression following retrobulbar bupivacaine: three case reports and literature review. Ophthalmic Surg 1987; 18: 768–771. 3323982

[4] Ahn JC Stanley JA . Subarachnoid injection as a complication of retrobulbar anesthesia. Am J Ophthalmol 1987; 103: 225–230. 3812625 10.1016/s0002-9394(14)74232-1

[5] Drysdale BD . Experimental subdural retrobulbar injection of anesthetic. Ann Ophthalmol 1984; 16: 716–718. 6497217

[6] Wittpenn JR Rapoza P Sternberg P , et al. Respiratory arrest following retrobulbar anaesthesia. Ophthalmology 1986; 86: 867–870. 10.1016/s0161-6420(86)33649-2 3763129

[7] Smith JI . Retrobulbar bupivacaine can cause respiratory arrest. Ann Ophthalmol 1982; 14: 1005–1006. 7181329

[8] Hessemer V . [German] Peribulbäranästhesie versus Retrobulbäranästhesie mit Fazialisblock. Klin Monatsbl Augenheilkd 1994; 204: 75–89. 8170098 10.1055/s-2008-1035503

[9] Loots JH Koorts AS Venter JA . Peribulbar anesthesia. J Cataract Refract Surg 1993; 19: 7276. 10.1016/s0886-3350(13)80286-6 8426327

[10] Aquavella JV . Comment (Limbal anesthesia for cataract surgery). Ophthalmic Surg 1990; 21: 26. 2325991

[11] Stewart MW Labrou FH . Local anesthetic for vitreoretinal surgery (Letter). Arch Ophtalmol 1993; 111: 161. 10.1001/archopht.1993.01090020015004 8431142

[12] Atkinson WS . Retrobulbar injection of anesthetic within the muscular cone. Arch Ophthalmol 1936; 16: 494–503.

[13] Castillo A Lopez-Abad C Marcias JM , et al. Respiratory arrest after 0.75% bupivacaine retrobulbar block. Ophthalmic Surg 1994; 25: 628–629. 7831008

[14] Cionni RJ Osher RH . Retrobulbar hemorrhage. Ophthalmology 1991; 98: 1017–1024. 1923350 10.1016/s0161-6420(91)32158-4

[15] Duguid IGM Claque CMP Thamby-Rajah Y , et al. Topical anaesthesia for phacoemulsification surgery. Eye 1995; 9: 456–459. 7498566 10.1038/eye.1995.106

[16] Hamilton RC . Brainstem anesthesia following retrobulbar blockade. Anesthesiology 1985; 63: 688–690. 4061924 10.1097/00000542-198512000-00022

[17] Knapp H . On cocaine and its use in ophthalmic and general surgery. Arch Ophtalmol 1884; 13: 402–448.

[18] Liu C Youl B Moseley I . Magnetic resonance imaging of the optic nerve in extremes of gaze. Implication for the positioning of the globe for retrobulbar anaesthesia. Br J Ophtalmol 1992; 76: 728–733. 10.1136/bjo.76.12.728 PMC504392 1486074

[19] Unsöld R Stanley JA De Groot J . The CT-topography of retrobulbar anaesthesia: anatomic-clinical correlation of complications and suggestion of a modified technique. Graefes Arch Klin Ophthalmol 1981; 217: 125–136. 10.1007/BF00418987 6912767

[20] Schönfeld CL Brinkschmidt T . [German] Hirnstammanästhesie mit Atemstillstand nach Retrobulbäranästhesie – Kasuistik mit Literurüberblick. Klin Monatsbl Augenheilkd 2000; 217: 130–132. 11022669 10.1055/s-2000-10397

[21] Wang Y-L Lan G-R Zou X , et al. Apnea caused by retrobulbar anesthesia: a case report. World J Clin Cases 2022; 10: 11646. 36387800 10.12998/wjcc.v10.i31.11646 PMC9649527

[22] Nanda T Ross L Kerr G . A case of brainstem anesthesia after retrobulbar block for globe rupture repair. Case Rep Anesthesiol 2021. DOI: 10.1155/2021/2619327 PMC8687828 34938580

[23] Kostadinov I . Brainstem anaesthesia after retrobulbar block. Open Medicine (Poland) 2019; 14: 287–291. 10.1515/med-2019-0025 PMC6419387 30886900

[24] Tolesa K . Brainstem anesthesia after retrobulbar block: a case report and review of literature. Ethiop J Health Sci 2016; 26: 589–594. 28450776 10.4314/ejhs.v26i6.13 PMC5389080

[25] Dettoraki M . Generalized seizures and transient contralateral hemiparesis following retrobulbar anesthesia: a case report. BMC Anesthesiol 2015. DOI: 10.1186/s12871-015-0084-y PMC4517554 26215739

[26] Jaichandran V . Brainstem anesthesia presenting as contralateral third nerve palsy following peribulbar anesthesia for cataract surgery. Acta Anaesthesiol Taiwan 2013; 51: 135–136. 24148744 10.1016/j.aat.2013.08.002

[27] Aranda Calleja MA Martínez Pueyo A Bellido Cuellar S , et al. Paresia de III par craneal y afectación troncoencefálica por difusión intradural de anestesia retrobulbar. Neurología 2011; 26: 563–564. 21715056 10.1016/j.nrl.2011.04.013

[28] Dahle JM . ED Treatment of brainstem anesthesia after retrobulbar block. Am J Emerg Med 2007; 25: 105–106. 17157702 10.1016/j.ajem.2006.05.030

[29] Pragt E . Delayed convulsion and brief contralateral hemiparesis after retrobulbar block. Reg Anesth Pain Med 2006; 31: 275–278. 16701195 10.1016/j.rapm.2006.01.004

[30] Chhokra R Cohen S Mohiuddin A , et al. It is just an eye case! delayed awakening after retro-bulbar block. J Anesth Clin Res 2013; 4: 334.

[31] Gunja N . Brainstem anaesthesia after retrobulbar block: a rare cause of coma presenting to the emergency department. Emerg Med Australas 2006; 18: 83–85. 16454780 10.1111/j.1742-6723.2006.00806.x

[32] George RB Hackett J . Bilateral hearing loss following a retrobulbar block. Can J Anesth 2005; 52: 1054–1057. 16326675 10.1007/BF03021604

[33] Long B Chavez S Gottlieb M , et al. Local anesthetic systemic toxicity: a narrative review for emergency clinicians. Am J Emerg Med 2022; 59: 42–48. ISSN 0735-6757. 35777259 10.1016/j.ajem.2022.06.017

[34] Mahan M Flor R Purt B . Local and regional anesthesia in ophthalmology and ocular trauma. StatPearls Publishing, 2023, [Updated 2023 May 7] Treasure Island (FL). PMID: 34662068. 34662068

[35] Williams DJ Walker JD . A nomogram for calculating the maximum dose of local anaesthetic. Anaesthesia 2014; 69: 847–853. 24820093 10.1111/anae.12679

[36] Taylor A , et al. Basic pharmacology of local anaesthetics. BJA Educ 2020; 20: 34–41. 33456928 10.1016/j.bjae.2019.10.002 PMC7808030

[37] Safety Committee of Japanese Society of Anesthesiologists. Practical guide for the management of systemic toxicity caused by local anesthetics. J Anesth 2019; 33: 1–8. 30417244 10.1007/s00540-018-2542-4

[38] Macfarlane AJR Gitman M Bornstein KJ , et al. Updates in our understanding of local anaesthetic systemic toxicity: a narrative review. Anaesthesia 2021; 76: 27–39. 10.1111/anae.15282 33426662

[39] Belov V Appleton J Levin S , et al. Large-volume intrathecal administrations: impact on CSF pressure and safety implications. Front Neurosci 2021; 15: 604197. 33935624 10.3389/fnins.2021.604197 PMC8079755

